# Limited Accuracy of Pan-Trk Immunohistochemistry Screening for *NTRK* Rearrangements in Follicular-Derived Thyroid Carcinoma

**DOI:** 10.3390/ijms23137470

**Published:** 2022-07-05

**Authors:** Elisabetta Macerola, Agnese Proietti, Anello Marcello Poma, Paola Vignali, Rebecca Sparavelli, Alessandro Ginori, Alessio Basolo, Rossella Elisei, Ferruccio Santini, Fulvio Basolo

**Affiliations:** 1 Department of Surgical, Medical, Molecular Pathology and Critical Area, University of Pisa, 56126 Pisa, Italy; elisabetta.macerola@for.unipi.it (E.M.); a.proietti@ao-pisa.toscana.it (A.P.); marcello.poma@med.unipi.it (A.M.P.); paola.vignali@phd.unipi.it (P.V.); r.sparavelli@studenti.unipi.it (R.S.); 2 Pathology Unit, USL Toscana Nord-Ovest, 54033 Carrara, Italy; alessandro.ginori@uslnordovest.toscana.it; 3 Department of Clinical and Experimental Medicine, University of Pisa, 56126 Pisa, Italy; alessio.basolo@med.unipi.it (A.B.); rossella.elisei@med.unipi.it (R.E.); ferruccio.santini@med.unipi.it (F.S.)

**Keywords:** thyroid cancer, *NTRK*, pan-Trk, immunohistochemistry

## Abstract

Patients with advanced thyroid cancer harboring *NTRK* rearrangements can be treated with highly effective selective inhibitors. Immunohistochemistry (IHC) analysis, to detect Trk protein expression, represents an appealing screening strategy for *NTRK* rearrangements, but its efficacy has been poorly explored in thyroid cancer. The aim of this study is to investigate the diagnostic utility of Trk IHC in the identification of *NTRK* rearrangements. A series of 26 follicular-derived thyroid tumors, positive for *NTRK* rearrangements, and 28 *NTRK* fusion-negative controls were retrospectively analyzed by IHC using the pan-Trk monoclonal antibody (clone EPR17341) on the Ventana system. Area under the curve (AUC), sensitivity and specificity were calculated by ROC analysis. Trk expression was detected in 25 samples, including 22 out of the 26 *NTRK*-rearranged (84.6%) and three out of 28 *NTRK*-negative samples (10.7%). Four out of twenty-six *NTRK*-rearranged thyroid tumors were negative for Trk expression (15.4%), all carrying the *ETV6/NTRK3* fusion. The AUC, sensitivity and specificity were 0.87, 0.85 and 0.89, respectively. A screening based on IHC analysis showed limited sensitivity and specificity in the identification of *NTRK*-rearranged tumors. Since falsely negative results could preclude the administration of effective targeted drugs, alternative detection strategies should be considered for thyroid cancer.

## 1. Introduction

Structural rearrangements involving neurotrophic tyrosine receptor kinase (*NTRK*) cause a constitutive activation of Trk proteins, which represents a driving event in several cancer types. The Food and Drug Administration (FDA) tumor-agnostic approval of selective inhibitors targeting *NTRK* genes expanded the treatment options for patients with advanced tumors carrying these alterations [1]. Besides the relevant clinico-therapeutic implications, many authors focused on the identification of optimal *NTRK* fusion detection strategies to be implemented in the laboratory practice.

In thyroid cancer, *NTRK* rearrangements can be found in 2–4% of adult patients, with no evident differences among well-differentiated, poorly differentiated and undifferentiated histotypes [2,3,4,5,6,7,8]. The frequency in pediatric patients with papillary thyroid carcinoma (PTC) is higher, ranging from 8 to 15% [9,10,11,12].

The evaluation of *NTRK* rearrangements can be performed with a variety of techniques at different levels, by using chromosomal locus-specific probes (FISH, fluorescent in situ hybridization), DNA and RNA sequencing (NGS, next-generation sequencing-based testing), fusion transcript detection (RT-PCR, reverse-transcription polymerase chain reaction, nanoString system) and protein expression analysis (IHC, immunohistochemistry). Each methodology presents its own advantages and limitations, in terms of analytical sensitivity and specificity, technical equipment, time of execution, required expertise, amount of biological material and costs [13,14,15,16].

To ensure the optimal management of biological material and laboratory resources with a reasonable turn-around time, several *NTRK* testing algorithms have been proposed, specifically focused on thyroid cancer. Some authors would recommend performing IHC testing first, and then: in the case of protein expression, confirmation by RT-PCR or FISH; in the case of negative IHC staining, NGS testing [14,17]. Other authors suggest that no confirmation is needed in the case of IHC positivity, whilst IHC negative cases should undergo further confirmation, only in the presence of morphological tumor features indicative of *NTRK* fusion [18]. Some authors would encourage the use of NGS tests, mainly targeted RNA-based panels [13,15,19]; otherwise, IHC can be performed as a screening technique: IHC-negative cases can be considered as truly negative, while IHC-positive samples should be further analyzed for confirmation [13,19]. This variability in *NTRK* testing recommendations is in part due to the rarity of *NTRK* rearrangements. This has likely caused difficulties in designing robust and effective testing algorithms. In particular, Trk IHC interpretation appears to be inconsistent: positivity is at times presented as an affordable indicator of the presence of a rearrangement, and at others as weak evidence of *NTRK* fusion. Indeed, the IHC expression pattern of Trk proteins can be highly variable depending on the type of tumor tissue and also on the specific fusion event (*NTRK* gene and fusion partner involved) [14]. Moreover, IHC testing presents an intrinsic technical variability across laboratories and can be subjected to inter-observer interpretation variations [20].

Although *NTRK* testing algorithms based on IHC screening have already been proposed, only a few studies focused on Trk expression in thyroid cancer have been conducted. Herein, we performed Trk IHC analysis in a series of *NTRK*-positive and *NTRK*-negative thyroid tumors, with the aim to evaluate the diagnostic efficacy of IHC screening and identify peculiar Trk expression patterns.

## 2. Results

### 2.1. Pan-Trk Immunohistochemistry in Thyroid Cancer

**Figure 1:**
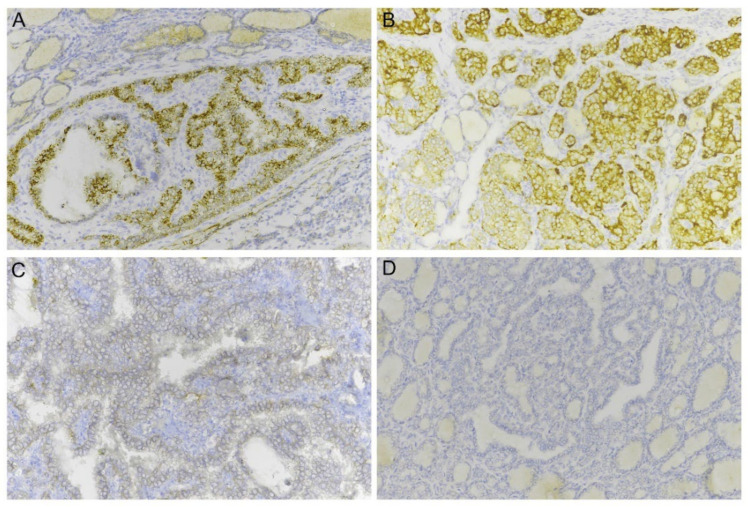
Pan-Trk IHC in papillary thyroid carcinoma. A strong granular cytoplasmic immunoreactivity is evident in neoplastic cells of a classical PTC carrying a *ETV6/NTRK3* rearrangement (**A**); strong immunoreactivity is clear and specific in cell membrane and cytoplasm of neoplastic cells in a case of *NTRK3*-rearranged classical PTC (**B**); weak and focal immunopositivity for pan-Trk in a case of classical PTC that was negative for *NTRK* rearrangements (**C**); no immunoreactivity is observed in a case of follicular variant PTC that was positive for the *ETV6/NTRK3* rearrangement (**D**).

A total of 25 samples out of 54 (46.3%) showed a positive Trk immunoreactivity, as shown in Table 1. In detail, IHC-positive cases included 22 out of the 26 *NTRK* fusion-positive (84.6%) and 3 out of the 28 *NTRK* fusion-negative samples (10.7%). The three control cases showing Trk proteins expression had *RET* fusion (classical PTC), *HRAS* point mutation (follicular variant PTC) and *NRAS* point mutation (local recurrence of PTC), respectively. In all IHC-positive samples, a signal was present in more than 10% of tumor cells. Signal intensity was mostly mild and strong (scores of 2+ and 3+) in 22 out of 25 cases (88%). Two out of the three IHC false-positive cases showed weak immunoreactivity (1+). The majority of samples showed cytoplasmic Trk expression (n = 20), with a granular pattern; in the remaining samples (n = 5), Trk staining was prevalently membranous (Figure 1).

The remaining 29 cases were negative for Trk expression (53.7%), including 4 out of 26 *NTRK*-rearranged cases which showed false-negative IHC staining (15.4%). These four discordant cases had all the same rearrangement, the *ETV6/NTRK3*, and all were PTCs (three classical and one follicular variant).

### 2.2. Pan-Trk Immunohistochemistry Test Performance

**Figure 2:**
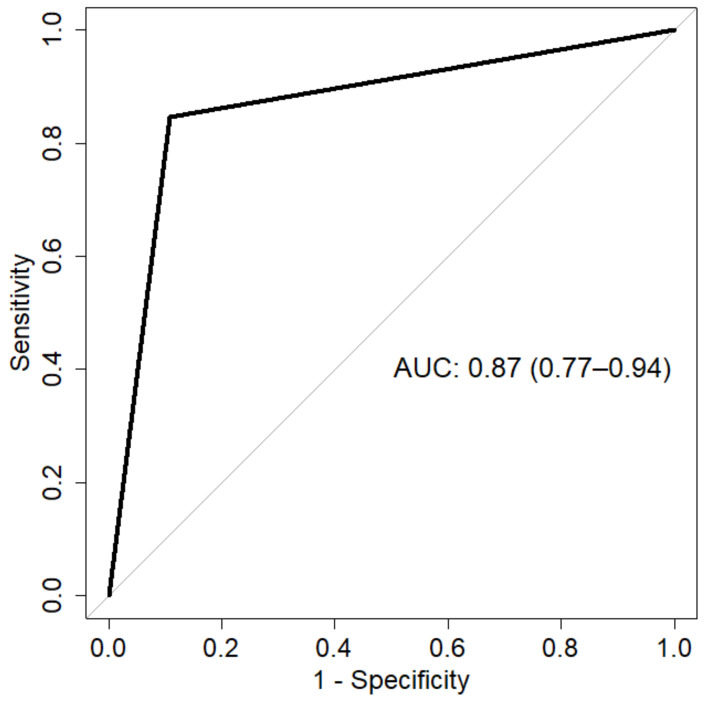
ROC analysis. The AUC represents the performance of pan-Trk IHC analysis in the identification of *NTRK* fusion-positive tumors.

By ROC analysis, Trk expression testing showed 0.87 area under the curve (AUC; 95% CI, 0.77–0.94) (Figure 2), 0.85 sensitivity (95% CI, 0.69–0.96), 0.89 specificity (95% CI, 0.75–1), 0.87 accuracy (95% CI, 0.78–0.94), 0.88 positive predictive value (PPV; 95% CI, 0.77–1) and 0.87 negative predictive value (NPV; 95% CI, 0.76–0.96) in identifying *NTRK*-rearranged tumors. The calculated sensitivity and specificity values are below 90% due to the presence of both false-positive and false-negative cases. As a consequence, the overall accuracy of pan-Trk testing in the identification of *NTRK* rearrangements is relatively low.

## 3. Discussion

In adult thyroid cancer, *NTRK* rearrangement is not common, with a prevalence of 2–4% [21]. However, in advanced-stage thyroid tumors, the availability of life-saving drugs targeting rearranged *NTRK* has made its testing mandatory.

Several testing algorithms have been proposed, but no broad consensus has been reached on the optimal analysis strategy. In particular, an approach based on the IHC analysis of Trk protein expression is recommended by several authors, as a rapid and cost-effective screening tool for *NTRK* rearrangements. One of the most widely used antibodies (pan-Trk monoclonal antibody, clone EPR17341) was optimized to detect a C-terminal portion common to TrkA, TrkB and TrkC proteins. A certain rate of false positivity could be expected, since the antibody cannot distinguish between the native protein expression and its chimeric forms.

It is known that the Trk staining pattern is highly variable according to several factors: the specific tumor tissue, the *NTRK* gene involved and the partner gene. Some studies have reported a relationship between the signal localization and the specific fusion event. For instance, a prevalently nuclear staining was observed exclusively in *ETV6/NTRK3* fusions cases [5,22]. In thyroid cancer, the described IHC signal pattern is generally cytoplasmic and/or membranous, with a cytoplasmic and nuclear staining in *NTRK3*-rearranged tumors [18,23,24]. In our study, the majority of Trk-positive cases presented a cytoplasmic staining.

Independently of the subcellular localization of signal, the pan-Trk antibody has demonstrated high levels of sensitivity and specificity for *NTRK* rearrangements in various cancer types [22,25,26,27]. In detail, Hechtman et al. reported a high level of concordance between pan-Trk testing and RNA-based NGS across 22 *NTRK* fusion-positive and 20 fusion-negative tumors of various histotypes, with one false-negative colorectal cancer sample carrying the *ETV6/NTRK3* fusion (specificity—100%; sensitivity—95.2%) [22]. In 79 pediatric mesenchymal tumors analyzed by Rudzinski and collaborators, pan-Trk IHC showed 98% specificity and 97% sensitivity in the identification of *NTRK* fusions [26]. Moreover, pan-Trk antibody testing showed good diagnostic performances in secretory carcinoma of the breast [27] and secretory carcinoma of the salivary gland [28].

On the other hand, there are studies highlighting that Trk IHC testing has important limitations. In a recent study conducted on lung carcinoma, the authors found that 12 out of 387 (3.1%) cases showed positive IHC staining; however, for NGS testing, all these cases were negative for rearrangements [29]. Across 327 samples from multiple cancer types, Koopman and colleagues reported 84% specificity and 77% sensitivity for pan-Trk IHC compared to RNA- and DNA-based NGS [30]; with regard to false-negative cases, 6 out of 29 *NTRK*-rearranged tumors showed a negative pan-Trk stain (20.7%).

In thyroid cancer, Lee and colleagues found that pan-Trk IHC showed high specificity but moderate sensitivity for *NTRK* fusion-driven PTCs, due to the presence of false-negative staining in 5 out of 12 *NTRK*-rearranged tumors (41.7%) [18]. Similarly, Gatalica and collaborators demonstrated that IHC testing for Trk proteins can be challenging. Among 70 thyroid carcinomas, the authors found four *NTRK3*-rearranged cases (5.7%), of which only two (50%) showed positive pan-Trk staining [24]. Solomon and colleagues detected 13 *NTRK* fusions among 571 thyroid carcinomas (2.3%); pan-Trk IHC analysis showed 100% specificity but lower sensitivity (82%) for *NTRK* rearrangements [5].

In our study, pan-Trk IHC testing did not show satisfying specificity (89%) nor sensitivity (85%). The four cases showing false-negative IHC results in our series were positive for rearrangements involving the *NTRK3* gene (*ETV6/NTRK3*). These findings are consistent with previous evidence indicating that pan-Trk reliability is poorer in *NTRK3*-rearranged tumors, compared with *NTRK1* and *NTRK2* genes [13,19]. With regard to false-positive cases, there are many possible causes: misinterpretation due to background signal; the overexpression of Trk proteins independent of structural rearrangements; non-specific antibody reaction; or cross-reaction. On the other hand, it is difficult to explain why, in some cases, the *ETV6/NTRK3* is associated with negative IHC staining. In our study, all *ETV6/NTRK3* rearrangements were detected at the RNA level, and thus the fusion transcript was sufficiently expressed to be measured. It is not known whether some biological factors could influence antibody reaction, or even the chimeric transcript translation; however, this would also affect the oncogenic potential of *NTRK3* fusion. To our knowledge, no specific studies have been conducted to address this issue.

Beyond these considerations, it must be highlighted that the *ETV6/NTRK3* is the most frequent structural rearrangement involving *NTRK* genes described in thyroid cancer [2,3,12,23]; therefore, a screening based on IHC testing could miss essential information in thyroid tumors. In fact, in our series, the IHC screening would have missed 4 out of 26 rearranged cases (15.4%). The *NTRK* testing algorithms that recommend using IHC analysis as a screening tool and NGS testing in the case of negative staining might overcome this poor performance in terms of sensitivity, allowing the recovery of eventual false-negative tumors. However, in practical terms, independently of the IHC results, samples should be further analyzed by a molecular test (i.e., RT-PCR, NGS) to exclude both false-positive and false-negative immunoreactions. Therefore, IHC-based testing does not represent an effective strategy in the screening of *NTRK* rearrangement in thyroid cancer.

This study presents some limitations. The sample size might appear too low to appropriately assess important diagnostic parameters, such as sensitivity and specificity. However, our sample series represents one of the largest ever reported including *NTRK*-rearranged thyroid tumors. In addition, differently from other studies focused on Trk IHC analysis, the negative cases included as controls were positive for other driver alterations, known to be mutually exclusive with *NTRK* rearrangements. Another limitation could be the lack of information of the fusion partner for 11 *NTRK*-rearranged tumors, due to the employed detection method (FISH, RT-PCR). Currently, the administration of drugs targeting rearranged *NTRK* is not related to the identification of the fusion partner; however, in the future, this aspect will likely be crucial to understanding whether the partner gene could influence not only IHC performance, but also treatment efficacy.

In conclusion, the recent approval of drugs targeting *NTRK*-rearranged tumors highlighted the necessity of developing new diagnostic algorithms to be applied in the molecular pathology setting. Our study demonstrated that using an IHC-based approach for the detection of Trk protein expression in thyroid cancer could present serious sensitivity issues. The diagnostic algorithm for testing *NTRK* rearrangements in this tumor model should include alternative analysis strategies, including in situ or nucleic acid-based detection methods.

## 4. Materials and Methods

### 4.1. Samples

A total of 54 thyroid tumors with available molecular profiles were selected from the archives of the Pathological Anatomy Unit of the University Hospital of Pisa. In detail, the case series was composed by 26 *NTRK* fusion-positive and 28 *NTRK* fusion-negative thyroid tumors, used as negative controls. To ensure *NTRK* negativity in control cases, besides a negative *NTRK* fusion test, only tumors carrying other driver genetic alterations were included. Cases were included only based on their molecular status, independent of eligibility for *NTRK*-targeted treatment. The most represented histological type was papillary thyroid carcinoma (PTC). Details on the sample series, including histo-pathological information, are shown in Table 2. All the experimental procedures were conducted on anonymous samples, according to the Declaration of Helsinki. Informed consent was waived due to the anonymous nature of the study. The study protocol received the institutional ethical committee approval (CEAVNO, protocol number 9989/2019).

*NTRK* status was assessed by different methodologies, as reported in Table 2. The most frequent fusion was the *ETV6/NTRK3*, detected in 13 out of 26 rearranged cases (50%). In all *ETV6/NTRK3* cases analyzed by NGS, the specific rearrangement involved exon 4 of *ETV6* and exon 14 of *NTRK3* (COSF1534). The employed NGS panel (Myriapod NGS Cancer Panel RNA, Diatech Pharmacogenetics, Iesi, AN, Italy) allowed the detection of the most common *NTRK* fusion variants described in cancer, 243 in *NTRK1*, 330 in *NTRK2*, and 154 in *NTRK3*. In case of FISH analysis, the fusion partner was unknown (break apart probes, ZytoLight SPEC NTRK1/NTRK3 Dual Color Break Apart Probe, Zytovision GmbH, Bremerhaven, Germany). For samples analyzed by RT-PCR (easyPGX Ready NTRK Fusion Kit, Diatech Pharmacogenetics), the employed methodology was unable to identify the specific partner gene of *NTRK1*, due to the presence of multiple probes in the same well. No *NTRK2*-positive tumors were present in this study; in fact, no *NTRK2* fusion has ever been detected in thyroid cancer [21]. Parts of positive cases were included in our previous study, where fusion testing was conducted by the nCounter system, and then confirmed by orthogonal techniques [12].

### 4.2. IHC Analysis

For each of the included cases, a 4 um-thick slice was obtained from FFPE tissue blocks for IHC analysis; the most representative tissue block, the same as previously employed for *NTRK* fusion detection, was used. The VENTANA pan-TRK (EPR17341) assay (Roche Diagnostics Spa, Monza, MB, Italy) was used to assess the expression of Trk proteins. In detail, this in vitro-validated assay enables the detection of C-terminal region of TrkA, TrkB and TrkC, which should be maintained in case of *NTRK1*, *NTRK2* and *NTRK3* rearrangements. All procedures were conducted according to the methodology protocol. A positive control (appendix tissue) was included in each experimental session, as recommended by the manufacturer.

IHC staining was interpreted by three qualified pathologists. Positivity was deemed in cases with signal above background in at least 1% of tumor cells, as indicated by the manufacturer. Signal intensity, percentage of positive cells and subcellular staining localization (membranous, cytoplasmic and nuclear) were recorded. In discordant cases, a consensus was reached by collegial discussion. Signal intensity was expressed as a score, from 1 to 3, corresponding to weak, mild and strong signals [31].

### 4.3. Data Analysis

The area under the curve (AUC), specificity, sensitivity, accuracy, positive predictive value (PPV) and negative predictive value (NPV) of IHC analysis for *NTRK* fusions were calculated by ROC analysis, along with related 95% confidence intervals (CI) by using 2000 bootstrap resampling. The analysis was performed following the procedures of the pROC R package v.1.18.0 in R environment (https://www.r-project.org, v.4.1.2; last accessed on 9 December 2021).

## Figures and Tables

**Table 1:** Characteristics of 25 thyroid tumors showing positive immunohistochemical Trk stain. The *NTRK* gene, the fusion partner gene (if known), the percentage of positive tumor cells, signal localization and intensity, and tumor histotype are reported. For samples positive for Trk expression but negative for *NTRK* rearrangements (N20, N24, N28), the driver alteration is indicated.

Sample Name	Driver Gene	Fusion Partner	Positive Cells (%)	Localization of Positive Signal	Signal Intensity	Tumor Histotype
P1	*NTRK1*	unknown	50	Cytoplasmic (granular)	2+	PTC—diffuse sclerosing variant
P2	*NTRK1*	unknown	50	Cell membrane	2+	PTC—classical type
P3	*NTRK1*	unknown	80	Cytoplasmic (granular)	3+	PTC—classical type
P4	*NTRK1*	unknown	60	Cell membrane	2+	PTC—classical type
P5	*NTRK1*	unknown	70	Cytoplasmic (granular)	2+	PTC—classical type
P6	*NTRK1*	*TPM3*	70	Cytoplasmic (granular)	3+	PDTC
P7	*NTRK3*	*ETV6*	40	Cytoplasmic (granular)	2+	PTC—follicular variant
P8	*NTRK3*	unknown	80	Cytoplasmic (granular)	2+	PTC—classical type
P11	*NTRK3*	unknown	60	Cell membrane	3+	PTC—follicular variant
P12	*NTRK3*	unknown	30	Cytoplasmic (granular)	3+	PTC—classical type
P13	*NTRK3*	unknown	70	Cytoplasmic	3+	PTC—classical type
P14	*NTRK3*	*ETV6*	20	Cytoplasmic (granular)	1+	PTC–solid variant
P15	*NTRK3*	*ETV6*	10	Cell membrane	2+	PTC—classical type
P17	*NTRK3*	*ETV6*	30	Cytoplasmic (granular)	3+	PTC—classical type
P18	*NTRK3*	unknown	30	Cytoplasmic (granular)	3+	PTC—classical type
P19	*NTRK3*	*ETV6*	40	Cytoplasmic (granular)	3+	PTC—classical type
P20	*NTRK3*	*ETV6*	30	Cytoplasmic (granular)	2+	PTC—classical type
P21	*NTRK3*	unknown	70	Cytoplasmic (granular)	3+	PTC—classical type
P22	*NTRK3*	*SQSTM1*	20	Cytoplasmic (granular)	3+	PTC—classical type
P24	*NTRK3*	*ETV6*	20	Cytoplasmic (granular)	2+	PTC—classical type
P25	*NTRK3*	*ETV6*	30	Cytoplasmic (granular)	3+	PTC—classical type
P26	*NTRK3*	*ETV6*	80	Cytoplasmic (granular)	3+	ATC
N20	*RET* fusion	/	10	Cell membrane	1+	PTC—classical type
N24	*HRAS* p.Q61K	/	15	Cytoplasmic (granular)	1+	PTC—follicular variant
N28	*NRAS* p.Q61R	/	70	Cytoplasmic (granular)	2+	PTC—metastasis

Abbreviations: PTC, papillary thyroid carcinoma; PDTC, poorly differentiated thyroid carcinoma; ATC, anaplastic thyroid carcinoma; IHC, immunohistochemistry.

**Table 2:** *NTRK*-rearranged thyroid tumors and *NTRK* fusion-negative controls. The methodology of *NTRK* fusion detection and the identity of the partner gene, if known, are reported. In cases negative for *NTRK* rearrangements, the detected driver genetic alteration is indicated. Histological diagnosis has been reported for each tumor. In cases of papillary thyroid carcinoma, the variant has been also indicated.

Sample Name	*NTRK* Gene	*NTRK* Status Assessed by	Fusion Partner	Non-*NTRK* Driver Alteration	Histology
P1	*NTRK1*	FISH	unknown	/	PTC—diffuse sclerosing variant
P2	*NTRK1*	RT-PCR	unknown	/	PTC—classical type
P3	*NTRK1*	FISH	unknown	/	PTC—classical type
P4	*NTRK1*	RT-PCR	unknown	/	PTC—classical type
P5	*NTRK1*	FISH	unknown	/	PTC—classical type
P6	*NTRK1*	NGS	*TPM3*	/	PDTC
P7	*NTRK3*	RT-PCR	*ETV6*	/	PTC—follicular variant
P8	*NTRK3*	FISH	unknown	/	PTC—classical type
P9	*NTRK3*	RT-PCR	*ETV6*	/	PTC—classical type
P10	*NTRK3*	RT-PCR	*ETV6*	/	PTC—follicular variant
P11	*NTRK3*	FISH	unknown	/	PTC—follicular variant
P12	*NTRK3*	FISH	unknown	/	PTC—classical type
P13	*NTRK3*	FISH	unknown	/	PTC—classical type
P14	*NTRK3*	RT-PCR	*ETV6*	/	PTC—solid variant
P15	*NTRK3*	RT-PCR	*ETV6*	/	PTC—classical type
P16	*NTRK3*	RT-PCR	*ETV6*	/	PTC—classical type
P17	*NTRK3*	RT-PCR	*ETV6*	/	PTC—classical type
P18	*NTRK3*	FISH	unknown	/	PTC—classical type
P19	*NTRK3*	RT-PCR	*ETV6*	/	PTC—classical type
P20	*NTRK3*	RT-PCR	*ETV6*	/	PTC—classical type
P21	*NTRK3*	FISH	unknown	/	PTC—classical type
P22	*NTRK3*	NGS	*SQSTM1*	/	PTC—classical type
P23	*NTRK3*	NGS	*ETV6*	/	PTC—classical type
P24	*NTRK3*	NGS	*ETV6*	/	PTC—classical type
P25	*NTRK3*	NGS	*ETV6*	/	PTC—classical type
P26	*NTRK3*	RT-PCR	*ETV6*	/	ATC
N1	/	FISH	/	*BRAF* p.V600E	PTC—classical type
N2	/	FISH	/	*BRAF* p.V600E	PTC—classical type
N3	/	FISH	/	*BRAF* p.V600E	PTC—classical type
N4	/	FISH	/	*BRAF* p.V600E	PTC—classical type
N5	/	FISH	/	*BRAF* p.V600E	PTC—classical type
N6	/	FISH	/	*BRAF* p.V600E	PTC—classical type
N7	/	FISH	/	*BRAF* p.V600E	PTC—classical type
N8	/	FISH	/	*NRAS* p.Q61R	PTC—follicular variant
N9	/	FISH	/	*RET* fusion	PTC—solid variant
N10	/	FISH	/	*RET* fusion	PTC—classical type
N11	/	FISH	/	*PPARG* fusion	PTC—follicular variant
N12	/	FISH	/	*NRAS* p.Q61K	PTC—follicular variant
N13	/	FISH	/	*RET* fusion	PTC—classical type
N14	/	FISH	/	*ALK* fusion	PTC—follicular variant
N15	/	FISH	/	*BRAF* p.V600E	PTC—classical type
N16	/	FISH	/	*RET* fusion	PTC—classical type
N17	/	FISH	/	*BRAF* p.V600E	PTC—classical type
N18	/	FISH	/	*BRAF* p.V600E	PTC—classical type
N19	/	FISH	/	*ALK* fusion	PTC—follicular variant
N20	/	NGS	/	*RET* fusion	PTC—classical type
N21	/	FISH	/	*BRAF* p.V600E	PTC—classical type
N22	/	FISH	/	*BRAF* p.V600E	PTC—classical type
N23	/	FISH	/	*BRAF* p.V600E	PTC—classical type
N24	/	FISH	/	*HRAS* p.Q61K	PTC—follicular variant
N25	/	FISH	/	*BRAF* p.V600E	PDTC
N26	/	RT-PCR	/	*BRAF* p.V600E	PTC—lymph node recurrence
N27	/	RT-PCR	/	*BRAF* p.V600E	PTC—local recurrence
N28	/	RT-PCR	/	*NRAS* p. Q61R	PTC—local recurrence

Abbreviations: PTC, papillary thyroid carcinoma; PDTC, poorly differentiated thyroid carcinoma; ATC, anaplastic thyroid carcinoma; FISH, fluorescent in situ hybridization; RT-PCR, reverse-transcription polymerase chain reaction; NGS, next-generation sequencing.

## Data Availability

All data generated or analyzed during this study are included in this published article.
